# Does food literacy influence healthy food choices? Findings from a cross-sectional study in Saudi Arabia

**DOI:** 10.3389/fnut.2026.1773427

**Published:** 2026-03-09

**Authors:** Hend Alhudhaif, Narmeen Shaikh, Noara Alhusseini

**Affiliations:** Department of Biostatistics, Epidemiology and Public Health, College of Medicine, Alfaisal University, Riyadh, Saudi Arabia

**Keywords:** food literacy, healthy food choices, nutrition education, public health, Saudi Arabia

## Abstract

**Background:**

Food literacy has emerged as an essential determinant of dietary behavior, encompassing the skills needed to access, understand, evaluate, and apply food- and nutrition-related information in daily life. Evidence examining food literacy and its relationship with healthy food choices among adults in Saudi Arabia remains limited.

**Objectives:**

This study aimed to assess food literacy levels and healthy food-choice priorities among adults living in Saudi Arabia and to examine the association among food literacy, food-quality priorities, and sociodemographic factors.

**Methods:**

A cross-sectional study was conducted using a survey that included sociodemographic variables, a validated food quality questionnaire assessing food choice priorities, and the Short Food Literacy Questionnaire (SFLQ). Descriptive statistics were used to summarize participant characteristics. Associations between food literacy, food choice priorities, and sociodemographic factors were examined using chi-square tests, Pearson correlation, and multivariable binary logistic regression analyses.

**Results:**

A total of 901 adults participated in the study. Higher food literacy was significantly associated with greater prioritization of health-related food attributes. Women and older adults demonstrated higher food literacy, whereas healthy food-choice priorities did not differ significantly by gender. Participants residing in the Western and Northern regions had higher odds of adequate food literacy than those in the Central region. Respondents with education up to high school were more likely to have adequate to excellent food literacy than those with higher education, while household income was not significantly associated with either food literacy or food choice priorities. Retired participants demonstrated healthier food-choice priorities than other employment groups.

**Conclusion:**

Food literacy is associated with healthier food choice priorities among adults in Saudi Arabia and varies across sociodemographic and regional groups. However, higher food literacy does not consistently translate into more nutritious choices, underscoring the influence of environmental and structural factors. Skill-based, contextually tailored food literacy interventions may support healthier dietary behaviors and help reduce diet-related health disparities.

## 1 Introduction

Unhealthy dietary patterns are a major contributor to the global burden of noncommunicable diseases, including obesity, cardiovascular disease, type 2 diabetes, and certain cancers (1). Suboptimal diet quality remains a leading modifiable risk factor for premature morbidity and mortality worldwide, underscoring the need for effective population-level strategies to promote healthier food choices (2). Although food availability and affordability strongly influence dietary behavior (3), increasing attention has focused on the individual-level skills required to navigate contemporary food environments. These environments are characterized by a wide range of food options, pervasive nutrition marketing, and often inconsistent or misleading dietary information, all of which can complicate healthy decision-making. Within this context, food literacy has emerged as a multidimensional construct that extends beyond basic nutrition knowledge (4, 5). Food literacy encompasses the skills needed to access, understand, evaluate, and apply food- and nutrition-related information to support healthy food selection and dietary practices in everyday life. By integrating functional, interactive, and critical competencies, food literacy influences food purchasing, preparation, and consumption across the life course (4, 5). Importantly, food literacy is increasingly recognized as a modifiable determinant of dietary behavior, rather than a fixed individual trait (6). Unlike sociodemographic factors such as age or income, food literacy can be strengthened through targeted education, skill-building, and supportive food environments, making it a promising lever for public health nutrition interventions aimed at bridging the gap between nutrition knowledge and actual food-choice behavior.

Growing empirical evidence supports the relevance of food literacy for dietary behavior. Higher food literacy has been associated with better dietary quality, more informed food-related decision-making, and healthier eating behaviors. Individuals with higher food literacy tend to consume greater amounts of fruits and vegetables, rely less on convenience and ultra-processed foods, demonstrate better adherence to dietary recommendations, and show stronger skills in interpreting nutrition labels and health claims (7–11). Beyond dietary intake, food literacy has also been linked to greater confidence and autonomy in food-related decision-making, including meal planning, food purchasing, and food preparation (12). Conversely, limited food literacy may increase vulnerability to misleading health claims, inconsistent nutrition messaging, and commercial influences within the food environment. Silva et al. (13) highlight that individuals are increasingly exposed to complex, sometimes contradictory, nutrition messages through product labeling, advertising, and digital media. In such contexts, food literacy plays a critical role in enabling individuals not only to access information but also to critically appraise its credibility and relevance and translate it into meaningful food-related decisions. Limited food literacy may therefore exacerbate confusion and misinterpretation of nutrition information, reducing the effectiveness of public health messaging and reinforcing the distinction between possessing nutrition knowledge and being able to apply it in real-world food-choice situations (13).

The Kingdom of Saudi Arabia is a critical, underexplored setting for examining food literacy and healthy food choices. Over recent decades, rapid urbanization, economic growth, and globalization have driven substantial changes in dietary patterns, characterized by increased consumption of energy-dense, highly processed foods and reduced intake of traditional diets rich in whole foods (14). These changes reflect a broader nutrition transition and have coincided with the rising rates of obesity and diet-related noncommunicable diseases in the Kingdom (15, 16). Recent national estimates indicate that the prevalence of obesity among adults (15 years and above) in Saudi Arabia has reached 23.1% (17), while the prevalence of diabetes continues to rise, placing a substantial and growing burden on the healthcare system (16).

The contemporary Saudi food environment is characterized by the widespread availability of fast food, aggressive marketing of processed products, increasing reliance on food delivery platforms, and time constraints associated with urban lifestyles, all of which shape food-choice behaviors (18–21). Cultural norms, social eating practices, and family-based food decision-making further influence dietary patterns (20). Many food outlets, especially fast-food places, in Saudi Arabia offer culturally adapted menus tailored to local tastes and preferences, integrating traditional flavors and familiar food items into commercially prepared meals (22). This localization of food offerings, combined with the availability of convenient, ready-to-eat foods that align with cultural expectations, has contributed to shifts in eating practices and reinforced preferences for convenience, palatability, and accessibility over nutritional quality. Collectively, these environmental and cultural factors play a central role in shaping dietary patterns and food choices across the Saudi population.

In response, Saudi Arabia has placed increasing emphasis on prevention, health promotion, and lifestyle modification, in alignment with national health transformation initiatives (23). However, the effectiveness of these strategies depends not only on access to healthy food options but also on individuals’ ability to interpret nutrition information, critically evaluate health messages, and translate guidance into everyday food choices. Recent evidence indicates that food and nutrition literacy represent emerging public health challenges in Saudi Arabia, with substantial gaps observed across multiple population groups. Studies focusing on Saudi youth have reported particularly concerning patterns, with adolescents ranking among the least nutritionally literate in the Arab region (24). Across both adolescent and student populations, approximately 40–45% of participants have been classified as having poor nutrition literacy, with male gender consistently associated with a higher risk (25, 26). Marked regional disparities have also been reported, including substantially higher odds of poor nutrition literacy among adolescents residing in the Northern Border region than those living in Riyadh (26). Evidence from adult populations reinforces these findings. Assessments of food-related knowledge among Saudi adults have yielded low average scores, with significant differences by gender and educational level (27). At a broader level, population-based studies suggest that approximately half of the Saudi population has low health literacy, with older age, lower income, and lower educational attainment identified as key risk factors (28). Collectively, these patterns indicate that limited literacy may disproportionately affect socioeconomically vulnerable groups, potentially contributing to persistent dietary inequalities. Notably, several studies highlight a disconnect between knowledge and behavior in the Saudi population. Although more than half of students were aware of the health consequences of excessive added sugar intake, only one-third reported actively reducing their consumption (29). Similarly, although fruits were widely recognized as important sources of vitamins and minerals, a substantial proportion of participants did not prefer them as snack alternatives to fast-food options (30). These findings suggest that awareness alone is insufficient to support consistent healthy food choices, underscoring the importance of applied skills such as food literacy.

Despite growing international evidence linking food literacy to healthier dietary behaviors, empirical research examining this relationship in Middle Eastern contexts, particularly in Saudi Arabia, remains limited. Nutrition-related research in Saudi Arabia has predominantly focused on nutrition knowledge, dietary intake patterns, and obesity prevalence, rather than on food literacy as a comprehensive applied skill set. Moreover, many existing studies have targeted specific subpopulations, such as students or adolescents, limiting their generalizability to the broader adult population. Evidence examining food literacy as a multidimensional construct encompassing the ability to access, evaluate, and apply food-related information, and its association with healthy food-choice priorities among Saudi adults, remains limited. This evidence gap is particularly relevant given the increasingly complex food environment in Saudi Arabia, characterized by aggressive food marketing, widespread availability of ultra-processed foods, and growing exposure to nutrition information through digital media. Understanding how food literacy varies across sociodemographic groups and how it relates to food choice priorities may provide critical insights for designing contextually appropriate, skill-based nutrition education and public health interventions. Therefore, this study aimed to assess food literacy levels and healthy food choices among adults in Saudi Arabia and to examine the associations between food literacy, food quality priorities, and key sociodemographic factors. By focusing on a nationally diverse adult population and conceptualizing food literacy as a multidimensional, applied skill set, this study provides novel empirical evidence to inform contextually appropriate nutrition education strategies and public health interventions in Saudi Arabia.

## 2 Methods

### 2.1 Study design, population, and eligibility criteria

A cross-sectional, web-based survey was conducted among adults residing in Saudi Arabia. The study aimed to assess food literacy levels and their association with healthy food choices among adults.

Adults aged 18 years or older residing in Saudi Arabia at the time of data collection were eligible to participate. Both Saudi and non-Saudi residents were included. Individuals younger than 18 years or those who did not provide informed consent were excluded from the study.

Based on an estimated adult population of approximately 25 million in Saudi Arabia, as reported by the General Authority for Statistics (31), a minimum required sample size of 385 participants was calculated at a 95% confidence level and a 5% margin of error. A total of 901 participants completed the survey and were included in the analysis.

### 2.2 Sampling and data collection

A convenience sampling approach was used. Participants were recruited through widely used social media platforms, including WhatsApp, X (formerly Twitter), and Telegram. The survey link was distributed through public and private groups to maximize reach across the country’s regions. As recruitment relied on online platforms, participation was more accessible to individuals who were digitally literate and actively engaged on social media. Participation was voluntary, and no financial or material incentives were provided.

Data were collected using an anonymous, self-administered Google Forms questionnaire. Before accessing the survey, participants were provided with an electronic information sheet describing the study objectives, procedures, confidentiality assurances, and the voluntary nature of participation. Electronic informed consent was obtained from all participants before survey initiation.

### 2.3 Survey instrument

#### 2.3.1 Questionnaire design

The questionnaire consisted of three main sections:

1. *Sociodemographic characteristics:* Participants reported their age, gender, nationality, region of residence in Saudi Arabia, educational attainment, employment status, and monthly income. For analytical purposes, Saudi Arabia was grouped into five geographical areas:
  - Central (Riyadh, Qassim)
  - Western (Makkah, Medina)
  - Eastern (Eastern Province)
  - Northern (Hail, Tabuk, Al-Jawf, Northern Borders)
  - Southern (Asir, Najran, Al-Baha, Jazan)
2. *Healthy food choices (food quality):* Healthy food choices were assessed using a five-item Food Quality questionnaire adapted from a previously published study (32). It is a validated instrument designed to measure functional, interactive, and critical aspects of food literacy. The SFLQ consists of 12 items. Each item was rated on a 5-point Likert scale (1 = strongly disagree to 5 = strongly agree). Item scores were summed to generate a total food quality score ranging from 5 to 25, with higher scores indicating healthier food choices. For analysis, food quality scores were categorized as:
  - Agree (healthier food choices): total score ≥15
  - Disagree (less healthy food choices): total score <15
3. *Food literacy assessment:* Food literacy was measured using the Short Food Literacy Questionnaire (SFLQ) (33). The SFLQ comprises 12 items assessing abilities in understanding, evaluating, and applying nutrition and food-related information. Total scores range from 0 to 52, with higher scores indicating greater food literacy. Food literacy levels were categorized based on a cutoff reported (34):
  - Adequate to excellent food literacy: total score ≥31
  - Inadequate to limited food literacy: total score <31

The SFLQ and Food Quality questionnaires rely on self-reported responses and assess perceived food literacy and food choice priorities rather than objective nutritional knowledge or actual dietary intake. As with all self-assessment tools, responses may be influenced by social desirability or overestimation of abilities.

#### 2.3.2 Validity and reliability

The Food Quality questionnaire was initially adapted by Alhusseini and Alqahtani (32) from previously validated instruments (35, 36). Content validity of the food quality instrument was established in the original study through a clinical dietitian’s review, and no modifications were made to the questionnaire’s wording or structure in the present study.

The Short Food Literacy Questionnaire (SFLQ) is a validated instrument (33) for adult populations and was adapted for use in the Saudi context.

To enhance validity, the questionnaire underwent pretesting before distribution. Face validity was further ensured by translating the survey from English into Arabic and confirming conceptual equivalence with a translator. The survey was administered in both Arabic and English to accommodate participants who are Arabic- and non-Arabic-speaking.

### 2.4 Statistical analysis

Data were entered and analyzed using Microsoft Excel 2010 and IBM SPSS Statistics for Windows, Version 25. Descriptive statistics were used to summarize participant characteristics and study variables. Categorical variables were presented as frequencies and percentages, while continuous variables (food quality and food literacy scores) were summarized using means and standard deviations.

Associations between sociodemographic variables and food quality and food literacy categories were examined using the Chi-square test. The relationship between total food literacy scores and total food quality scores was assessed using Pearson’s correlation coefficient.

To identify factors independently associated with healthier food choices, binary logistic regression analysis was performed. Food quality (agree vs. disagree) was the dependent variable, and food literacy level and relevant sociodemographic variables were independent predictors. Results were reported as odds ratios (ORs) with 95% confidence intervals (CIs). A two-sided *p*-value <0.05 was considered statistically significant.

### 2.5 Ethical considerations

Ethical approval was obtained from the Institutional Review Board of Alfaisal University (Approval No. IRB-20208). Participation was voluntary, and participants could withdraw at any time. No identifiable information was collected, and all responses were anonymized and used exclusively for research purposes.

## 3 Results

### 3.1 Participant characteristics

A total of 901 adults residing in Saudi Arabia completed the online survey. Sociodemographic characteristics (gender, age group, relationship status, nationality, education level, employment status, household monthly income, and region of residence) are summarized in Table 1.

**Table 1:** Demographic characteristics of the respondents (*N* = 901).

Variable	Category	*n*	%
Gender	Male	444	49%
	Female	457	51%
Age group (years)	18–29	305	34%
	30–39	248	28%
	40–49	178	20%
	50–59	117	13%
	60+	53	6%
Relationship Status	Single	330	37%
	Married	474	53%
	Divorced, separated, or widowed	79	9%
	Rather not say	18	2%
Nationality	Saudi	752	83%
	Non-Saudi	149	17%
Educational level	High school or less	188	21%
	Bachelors	554	61%
	Higher education (Master’s or PhD)	159	18%
Employment status	Employed	454	50%
	Unemployed	320	36%
	Business Owner	58	6%
	Retired	69	8%
Income household monthly (SAR)	9,999 or less	262	29%
	10,000 – 19,999	260	29%
	20,000 – 29,999	118	13%
	30,000 or more	71	8%
	I prefer not to answer	190	21%
Region of Saudi Arabia	Central	538	60%
	Western	111	12%
	Eastern	100	11%
	Northern	83	9%
	Southern	69	8%

### 3.2 Food literacy levels

Based on the Short Food Literacy Questionnaire (SFLQ), participants were classified into adequate–excellent (total score ≥31) and inadequate-to-limited food literacy (total score <31) categories. The majority of participants demonstrated adequate to excellent food literacy (80%). Responses to individual items of the Short Food Literacy Questionnaire (SFLQ) are presented in Supplementary Tables S1, S2. Overall, participants reported higher agreement with items related to understanding nutrition information, recognizing healthy food choices, and applying nutrition knowledge in daily food selection. In contrast, comparatively lower agreement was observed for items related to judging the credibility of nutrition information and assessing long-term health impacts of dietary habits.

Food literacy levels differed significantly by gender, with a higher proportion of females classified as having adequate–excellent food literacy compared with males (*p* = 0.005). Significant differences were also observed across age groups (*p* = 0.015), relationship status (*p* = 0.017), and region of residence (*p* = 0.004). The highest proportion of adequate–excellent food literacy was observed among participants aged 60 years and older and among those residing in the Western and Northern regions. Associations between food literacy levels and sociodemographic characteristics are shown in Table 2.

**Table 2:** Demographic variables vs. food literacy levels.

Food literacy level (SFLQ)						
Variable	Category	Adequate-excellent (31+)		Inadequate-limited food literacy (<31)		*p*-value
		*n*	%	*n*	%	
Gender	Male	338	76%	106	24%	0.005
	Female	382	84%	75	16%	
Age	18–29	244	80%	61	20%	0.015
	30–39	205	83%	43	17%	
	40–49	154	87%	24	13%	
	50–59	99	85%	18	15%	
	60+	48	91%	5	9%	
Relationship status	Single	248	75%	82	25%	0.017
	Married	387	82%	87	18%	
	Divorced, separated, or widowed	69	87%	10	13%	
Nationality	Saudi	595	79%	157	21%	0.18
	Non-Saudi	125	84%	24	16%	
Education level	High School or less	153	81%	35	19%	0.14
	Bachelors	449	81%	105	19%	
	Higher education (Master’s or PhD)	118	74%	41	26%	
Employment status	Employed	347	76%	107	24%	0.054
	Unemployed	264	83%	56	17%	
	Business owner	49	84%	9	16%	
	Retired	60	87%	9	13%	
Household monthly income (SAR)	9,999 or less	197	75%	65	25%	0.26
	10,000 - 19,999	213	82%	47	18%	
	20,000 - 29,999	95	81%	23	19%	
	30,000 or more	58	82%	13	18%	
	I prefer not to answer	157	83%	33	17%	
Region of Saudi Arabia	Central	411	76%	127	24%	0.004
	Western	99	89%	12	11%	
	Eastern	78	78%	22	22%	
	Northern	74	89%	9	11%	
	Southern	58	84%	11	16%	

Binary logistic regression analysis identified several factors independently associated with adequate–excellent food literacy. Female participants had higher odds of having adequate to excellent food literacy than males. Participants aged 30–39 years and 60 years or older had higher odds compared with those aged 18–29 years. Regional differences remained significant, with higher odds observed among participants residing in the Western and Northern regions compared with the Central region. Results of the regression analysis are presented in Table 3.

**Table 3:** Logistic regression for variables associated with having adequate to excellent food literacy levels.

Variable	Category	*n*	OR	Lower confidence interval	Upper confidence interval	*p*-value
Gender	Male	338	(Ref)			
	Female	382	1.82	1.26	2.60	**0.001**
Age	18–29	244	(Ref)			
	30–39	205	1.61	1.01	2.58	**0.048**
	40–49	154	1.62	0.92	2.84	0.096
	50–59	99	1.59	0.81	3.12	0.181
	60+	48	3.62	1.21	10.82	**0.021**
Relationship status	Single	248	(Ref)			
	Married	387	1.02	0.66	1.57	0.94
	Divorced, separated, or widowed	69	1.20	0.53	2.63	0.68
Nationality	Saudi	595	(Ref)			
	Non-Saudi	125	1.37	0.81	2.25	0.25
Education level	Higher education (Master’s or PhD)	153	(Ref)			
	Bachelors	449	1.55	0.89	2.79	0.12
	High School or less	118	1.89	1.05	2.55	**0.03**
Employment status	Employed	347	Ref			
	Unemployed	264	1.31	0.90	2.03	0.14
	Business owner	49	1.46	0.65	3.35	0.36
	Retired	60	1.61	0.67	3.80	0.29
Region of Saudi Arabia	Central	411	(Ref)			
	Western	99	2.52	1.32	4.86	**0.01**
	Eastern	78	1.10	0.65	1.92	0.70
	Northern	74	2.88	1.33	6.41	**0.01**
	Southern	58	1.61	0.81	3.26	0.17

Variable(s) entered on step 1: gender, age, relation, nationality, education level, employment status, and region of Saudi Arabia. Bold values indicate statistically significant associations (*p* < 0.05).

### 3.3 Healthy food choices (food quality)

Healthy food choices were assessed using the Food Quality score and categorized into agree and disagree groups. Item-level responses for the Food Quality questionnaire are shown in Supplementary Table S3. The majority of participants chose ‘strongly agree’ with items emphasizing the health effects of food, freshness, and healthy food properties. A substantial portion of participants also agreed that items prioritizing long shelf life and food cost over nutritional quality were more important.

Differences in agreement on food quality were observed across several sociodemographic variables. Significant associations were identified with nationality and employment status, with Saudi nationals and retired participants more likely to endorse healthier food-choice priorities. These associations are presented in Table 4.

**Table 4:** Demographic variables vs. food quality awareness category.

Food quality group						
Variable	Category	Disagree (<15)		Agree (15+)		*p*-value
		*n*	%	*n*	%	
Gender	Male	90	20%	354	80%	0.22
	Female	78	17%	379	83%	
Age	18–29	66	22%	239	78%	0.29
	30–39	38	15%	210	85%	
	40–49	36	20%	142	80%	
	50–59	21	18%	96	82%	
	60+	7	13%	46	87%	
Relationship status	Divorced, separated, or widowed	15	19%	64	81%	0.31
	Single	69	21%	261	79%	
	Married	79	17%	395	83%	
Nationality	Non-Saudi	48	32%	101	68%	<0.001
	Saudi	120	16%	632	84%	
Education	High school or less	38	20%	150	80%	0.15
	Bachelors	93	17%	461	83%	
	Higher education	37	23%	122	77%	
Employment	Business Owner	15	26%	43	74%	0.001
	Unemployed	75	23%	245	77%	
	Employed	74	16%	380	84%	
	Retired	4	6%	65	94%	
Household income (SAR)	9,999 or less	58	22%	204	78%	0.33
	10,000 – 19,999	43	17%	217	83%	
	20,000 – 29,999	23	19%	95	81%	
	30,000 or more	15	21%	56	79%	
	Prefer not to answer	29	15%	161	85%	
Regions	Central	18	22%	65	78%	0.47
	Northern	18	26%	51	74%	
	Southern	19	17%	92	83%	
	Western	18	18%	82	82%	
	Eastern	95	18%	443	82%	

In multivariable analysis, Saudi nationality was independently associated with a higher odds of agreeing with food quality than non-Saudi nationality. In addition, retired participants had higher odds of agreement on food quality than business owners. Results of the binary logistic regression model for healthy food choices are shown in Table 5.

**Table 5:** Logistic regression for variables associated with food quality.

Variable	Category	OR	Lower confidence interval	Upper confidence interval	*p*-value
Nationality	Saudi	(Ref)			
	Non-Saudi	2.27	1.52	3.41	**<0.001**
Education level	Higher education (Master’s or PhD)	(Ref)			
	High School or less	1.31	0.76	2.28	0.33
	Bachelors	1.54	0.98	2.41	0.06
Employment	Business owner	(Ref)			
	Unemployed	0.93	0.48	1.81	0.84
	Employed	1.50	0.77	2.90	0.23
	Retired	4.45	1.36	14.53	**0.013**

Bold values indicate statistically significant associations (*p* < 0.05).

### 3.4 Association between food literacy and healthy food choices

**Figure 1:**
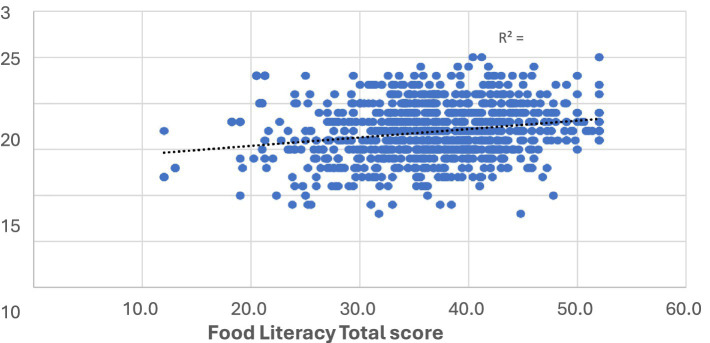
A weak but statistically significant positive correlation (*r* = 0.22, *p* < 0.001) between total food literacy and total food quality scores.

A positive correlation was observed between total food literacy scores and total food quality scores. Pearson’s correlation analysis showed a statistically significant association (r = 0.22, *p* < 0.001), indicating that higher food literacy was associated with healthier food choices. The relationship between food literacy and food quality scores is illustrated in Figure 1.

## 4 Discussion

This study provides novel insights into food literacy and healthy food choice priorities among adults in Saudi Arabia. The findings demonstrate that individuals with higher food literacy were more likely to prioritize health-related attributes when making food choices, consistent with international evidence linking higher food and nutrition literacy to improved diet quality, greater use of nutrition labels, and more informed food-related decisions (7–12). These findings align with conceptual frameworks that characterize food literacy as an applied skill set that enables individuals to translate nutrition information into everyday practice, particularly within complex food environments characterized by abundant choice, intensive marketing, and inconsistent nutrition messaging (4, 5, 13). Importantly, this association suggests that food literacy, as an applied skill set, may contribute to prioritizing health-related food attributes, reinforcing its potential as a modifiable target for promoting healthier eating behaviors. However, the magnitude of this association was modest (r = 0.22), indicating that while food literacy contributes meaningfully to food-choice priorities, other factors, including environmental, structural, and cultural influences, also play a substantial role. Interpreting these findings therefore requires careful consideration of the unique socio-cultural, economic, and policy context of Saudi Arabia, within which food literacy operates as a context-dependent rather than purely individual determinant of dietary behavior.

Gender differences in food literacy were evident, with women demonstrating higher levels than men. This is consistent with previous research: a national study of Saudi parents reported that fathers were 2.4 times as likely as mothers to exhibit poor food literacy (37), and similar trends have been reported internationally (38). One plausible explanation is that women in Saudi Arabia have traditionally borne primary responsibility for household food selection and preparation, which may foster greater practical engagement with food-related information and skill development. Despite higher food literacy among women, no significant gender difference was observed in self-reported food-choice priorities; the majority of both men and women reported agreement with health-oriented food attributes. This pattern may partly reflect limited variation in self-reported priorities. It also suggests that structural and environmental factors may shape food-choice priorities independently of food literacy levels. In Saudi Arabia, the widespread availability and affordability of ready-made meals, combined with the growing reliance on food delivery applications that emphasize convenience, speed, and palatability over nutritional quality (18–21, 39, 40), may further constrain the extent to which food literacy influences everyday food-choice priorities. Additionally, the pervasive marketing of energy-dense, processed food products across digital and traditional media channels creates a food environment in which individuals may choose less healthy options (41, 42). These findings highlight opportunities for targeted interventions, particularly through culturally appropriate approaches, such as workplace-based nutrition initiatives or male-focused food-skills programs.

Saudi nationality was independently associated with healthier food-choice priorities; however, this finding should be interpreted cautiously, given the relatively small proportion of non-Saudi participants (17%) compared with their representation in the national population. Non-Saudi residents who participated may not reflect the broader expatriate population, which encompasses diverse socioeconomic, linguistic, and cultural backgrounds. Nevertheless, the observed difference may partly reflect greater familiarity among Saudi participants with traditional dietary practices, such as home-prepared meals centered on rice, vegetables, legumes, and dates (43), which tend to align more closely with health-oriented food choices. Saudi participants may also benefit from greater exposure to national nutrition campaigns and public health messaging, which are predominantly delivered in Arabic through Saudi media channels, government healthcare services, and social media platforms to reach the general public. It should be noted, however, that key government health information and digital platforms are available in multilingual formats. Non-Saudi residents may still face challenges adapting their dietary habits to the local food environment, particularly when culturally familiar healthy food options are less available or are expensive, potentially increasing reliance on commercially prepared and convenience foods. Future research with a more balanced and representative sampling of non-Saudi residents is needed to examine this association more thoroughly.

Several additional sociodemographic patterns emerged in relation to food literacy. Contrary to common assumptions, higher formal educational attainment was not consistently associated with higher food literacy. Participants with a high school education or less had significantly higher odds of adequate food literacy than those with postgraduate degrees, whereas no significant difference was observed among those with bachelor’s degrees. Similar observations have been reported among Saudi populations, in which substantial proportions of university-educated individuals still exhibit limited food literacy (37). This finding reinforces the distinction between general academic education and applied food-related competencies. Individuals with lower levels of formal education may have greater direct engagement in food purchasing, preparation, and household food management, thereby fostering practical food literacy skills through daily experience. Conversely, individuals with higher educational attainment may face greater time constraints associated with demanding professional roles, which may reduce hands-on engagement with food-related tasks and increase reliance on convenience and commercially prepared foods. These patterns suggest that food literacy among the Saudi population may be shaped primarily through informal, experiential channels rather than formal academic instruction. Household income, in contrast, was not significantly associated with food literacy or food-choice priorities in this study. However, the absence of an income gradient in food literacy does not imply the absence of socioeconomic inequalities in diet quality, as food literacy and economic access represent distinct but interacting dimensions of healthy eating. While individuals across income groups may possess comparable food literacy skills, their ability to act on these skills may still be constrained by food prices, availability, and household resources. Prior research has shown that lower-income households may prioritize food quantity and cost over quality, limiting opportunities to make healthy food choices (44), and that food literacy alone is insufficient to overcome structural and economic constraints that shape dietary behavior (45). Future research should therefore examine how food literacy interacts with income, food security, and local food environments to shape actual dietary behaviors.

Our analysis also revealed regional disparities in food literacy within Saudi Arabia. Participants from the Western and Northern regions had significantly higher odds of adequate-to-excellent food literacy than those in the Central region (which includes the capital, Riyadh), indicating substantial geographic variation shaped by region-specific contextual factors. While previous studies have documented regional differences in nutrition literacy among Saudi adolescents (26), the present findings suggest that urbanization and service concentration alone do not necessarily translate into higher applied food literacy. The Western region encompasses Makkah and Medina, cities characterized by high cultural diversity due to the annual pilgrimage, which may increase exposure to diverse food practices and health-related awareness campaigns associated with pilgrimage health services (46, 47). The Northern regions may retain stronger adherence to traditional food practices and local food systems, supporting applied food literacy through direct engagement with food sourcing and preparation. The Central region, despite its concentration of healthcare infrastructure and educational institutions, also has the highest density of fast-food outlets, food delivery services, and commercial food marketing (48), which may paradoxically contribute to lower food literacy by normalizing convenience-oriented consumption patterns. Despite regional differences in food literacy, no significant variation was observed in food-choice priorities, with the majority of participants across all regions reporting agreement with health-oriented food attributes. This uniformity may partly reflect a ceiling effect in self-reported food-choice priorities, limiting the ability to detect meaningful regional differences. Nonetheless, the disconnect between regional variation in food literacy and the relative uniformity in reported food-choice priorities suggests that self-reported prioritization of healthy eating may not fully capture the influence of food literacy on actual dietary decision-making, and that contextual factors such as food availability, marketing exposure, and cost likely play an important role in shaping food-choice behavior. These findings emphasize the importance of region-specific strategies that address not only food literacy gaps but also the environmental conditions that enable or constrain the application of food literacy in everyday food choices.

Age and employment-related differences further underscore the complexity of food literacy and food choice behavior. Older adults and individuals aged 30–40 years demonstrated higher food literacy levels than younger participants, suggesting that food literacy may increase with cumulative life experience and prolonged engagement in food-related decision-making. Individuals in midlife may also be more motivated to seek and apply nutrition information due to increasing health awareness or the onset of diet-related conditions. Employment status was also associated with food choice priorities. Retired participants reported healthier food-choice priorities than other employment groups, despite comparable food literacy levels; however, this finding should be interpreted cautiously, given the small sample size (*n* = 69) and the wide confidence interval. Consistent with previous studies identifying time pressure and work demands as key barriers to healthy eating among working adults (49, 50), time availability, routine stability, and reduced work-related constraints may enable healthier food-choice priorities in retirees. In Saudi Arabia, government-sector employees typically retire at age 60 and often maintain stable household incomes through pension systems. This financial stability, combined with increased time for meal planning, food preparation, and deliberate food selection, may create particularly favorable conditions for translating food literacy into healthier food-choice priorities, a pattern that may be less pronounced in contexts where retirement is associated with income reduction.

From a public health perspective, these findings carry important implications for nutrition policy and practice in Saudi Arabia and are directly relevant to ongoing policy efforts, including the Saudi Food and Drug Authority’s mandatory calorie-labeling regulations for restaurants and cafés (51), the Health Sector Transformation Program under Vision 2030 (23), and the Quality of Life Program’s (52) emphasis on promoting healthy lifestyles. While these initiatives represent important structural interventions, the present findings suggest that their effectiveness may be enhanced by complementary food literacy programs that equip individuals with the skills to interpret and act on the information provided through such regulatory measures. For example, calorie labeling may have a limited impact among individuals with low food literacy who lack the ability to contextualize caloric information within their overall dietary patterns. Integrating food literacy into nutrition education initiatives across schools, community programs, healthcare settings, and digital platforms may strengthen individuals’ capacity to make informed food choices in real-world contexts. The observed sociodemographic patterns further suggest that food literacy interventions should be targeted, with particular attention to males, younger adults, and specific regions, to reduce disparities. Future research should employ longitudinal and intervention designs to examine causal pathways between food literacy and dietary behavior and incorporate objective measures of diet quality and food security. Qualitative approaches may also help elucidate how individuals interpret nutrition information and navigate competing food priorities. Together, such efforts would support the development of scalable, contextually appropriate strategies to improve food literacy and promote healthier eating across the Saudi population.

### 4.1 Strengths and limitations

This study has several notable strengths. First, it is one of the few population-based studies to examine food literacy and healthy food-choice priorities among adults in Saudi Arabia, thereby addressing a vital evidence gap in the regional literature. The inclusion of participants from multiple regions of the Kingdom enhances the geographic scope of the findings and allows for the identification of meaningful regional variations in food literacy. In addition, the relatively large sample size provides adequate statistical power to detect associations across key sociodemographic groups. Another strength is the use of validated measurement instruments, which enhances the study’s internal validity and facilitates comparison with international research. Furthermore, the analytic approach incorporated multivariable regression models, enabling examination of independent associations among food literacy, food choice priorities, and sociodemographic factors while adjusting for potential confounders. Also, evaluating food literacy alongside food quality priorities provides a more comprehensive understanding of food-related priorities.

Despite these strengths, several limitations should be considered when interpreting the findings. The cross-sectional design precludes causal inference. The use of convenience sampling and online data collection may have introduced selection bias and may also limit the generalizability of the findings. Participants were more likely to be younger adults, to have higher educational attainment, and to be digitally literate and engaged with online platforms. As a result, groups with limited internet access, lower digital literacy, or lower educational levels may be underrepresented, and overall food literacy levels may be overestimated. Additionally, non-Saudi residents were underrepresented relative to their share of the national population, and participants were likely more digitally literate and socially connected than the broader expatriate population, which limits the generalizability of nationality-related findings. Moreover, although participants were recruited from multiple regions, some regions were overrepresented relative to others, potentially influencing regional comparisons. Another limitation is that food-choice priorities were assessed rather than objective dietary intake data being collected. While prioritization of health-related food attributes provides valuable insight into decision-making processes, it may not fully capture actual eating behaviors or diet quality. Similarly, household income and food security were not assessed using detailed, standardized food-security instruments, limiting the ability to fully explore socioeconomic constraints on dietary behavior. Moreover, environmental influences such as food availability, pricing, and marketing exposure were not directly measured. Also, although the Short Food Literacy Questionnaire captures multiple dimensions of food literacy, it may not fully account for cultural and contextual factors specific to the Saudi food environment, such as traditional dietary practices or family-based food decision-making. Another important limitation is the reliance on self-reported questionnaires to assess food literacy and food choice priorities. Although the SFLQ and Food Quality questionnaires are validated instruments and widely used in research, they capture perceived abilities and priorities rather than objectively measured nutritional knowledge or dietary behavior. Respondents may overestimate their knowledge or provide socially desirable responses, particularly for items related to nutrition recommendations or healthy eating practices. In addition, response fatigue may influence self-assessment in longer questionnaires. These limitations should be considered when interpreting the findings. Future research should incorporate objective, performance-based measures of food literacy, such as knowledge-verification questions, label-interpretation tasks, or dietary assessment tools, to further validate and refine food literacy measurement in the Saudi context.

## 5 Conclusion

This study demonstrates that food literacy is positively associated with healthier food-choice priorities among adults in Saudi Arabia, highlighting its role as a critical and modifiable determinant of dietary decision-making. Meaningful sociodemographic and regional variations were observed, and higher food literacy did not consistently translate into healthier choices, reflecting the influence of broader environmental and structural conditions. These findings underscore the need for public health strategies that extend beyond information provision toward skill-based, context-responsive nutrition education. Strengthening food literacy across the life course, while addressing practical constraints such as time availability and the surrounding food environment, may support more effective and equitable improvements in diet quality and reduce the burden of diet-related noncommunicable diseases in Saudi Arabia. Embedding food literacy initiatives within the Kingdom’s socio-cultural and policy landscape will be essential to ensure their effectiveness, scalability, and long-term public health impact.

## Data Availability

The raw data supporting the conclusions of this article will be made available by the authors upon reasonable request.
