# Supplementary material for: Does food literacy influence healthy food choices? Findings from a cross-sectional study in Saudi Arabia
Source: Front Nutr. 2026 Mar 9;13:1773427. doi: 10.3389/fnut.2026.1773427 (PMC13006305; doi:10.3389/fnut.2026.1773427)
Supplement: Supplementary file 1 [file Data_Sheet_1.PDF]

Supplementary material

1. Supplementary Table S1

Table S1. SFLQ – Knowledge about Saudi Recommendations about Nutrition and understanding of Nutritional Information from different Sources

		Strongly Agree	Agree	Disagree	Strongly Disagree	I do not have experience with these issues	
When I have questions on healthy nutrition, I know where I can find information	N	264	389	130	29	89	
	%	29%	43%	14%	3%	10%	
I know the official Saudi recommendations about fruit and vegetable consumption	N	202	331	285	83	0	
	%	22%	37%	32%	9%	0%	
I know the official Saudi recommendations about salt intake	N	180	322	304	95	0	
	%	20%	36%	34%	11%	0%	
In general, how well do you understand the following types of nutritional information:		Very Good	Good	Okay	Bad	Very Bad	I do not understand
Nutrition information leaflets	N	245	262	233	72	28	61
	%	27%	29%	26%	8%	3%	7%
Food label information	N	276	299	205	62	21	38
	%	31%	33%	23%	7%	2%	4%
Social media verified channels on nutrition	N	219	278	203	92	36	73
	%	24%	31%	23%	10%	4%	8%

Oral recommendations regarding nutrition from professionals	N	239	267	245	75	31	44
	%	27%	30%	27%	8%	3%	5%
Nutrition advice from family members or friends	N	264	296	235	47	23	36
	%	29%	33%	26%	5%	3%	4%

2. Supplementary Table S2

Table S2. SFLQ – Evaluation of Media and Dietary Information

	Very Good	Good	Bad	Very Bad
How easy is it for you to judge if media information on nutritional issues can be trusted	207	434	192	68
	23%	48%	21%	8%
Commercials often relate foods with health. How easy is it for you to judge if the presented associations are appropriate or not	215	355	241	90
	24%	39%	27%	10%
How easy is it for you to evaluate if a specific food is relevant for a healthy diet	269	426	154	52
	30%	47%	17%	6%
How easy is it for you to evaluate the longer-term impact of your dietary habits on your health	233	363	252	53
	26%	40%	28%	6%

3. Supplementary Table S3

Table S3. Responses to Food Quality Questions (N=901)

	Strongly Agree	Agree	Neutral	Disagree	Strongl y Disagre e

I prefer a long shelf-life food items that take long time to expire such as canned food	186<br>21%	227<br>25%	216<br>24%	213<br>24%	59<br>7%
I prefer a lower cost	181<br>20%	268<br>30%	242<br>27%	170<br>19%	40<br>4%
I am very careful about the health effects of the food products	316	341	145	82	17
	35%	38%	16%	9%	2%
I am very careful about the freshness	420<br>47%	312<br>35%	99<br>11%	57<br>6%	13<br>1%
I choose products according to calories and healthy properties	226	242	251	132	50
	25%	27%	28%	15%	6%
